# Minimum number of nights for reliable estimation of habitual sleep using a consumer sleep tracker

**DOI:** 10.1093/sleepadvances/zpac026

**Published:** 2022-08-31

**Authors:** TeYang Lau, Ju Lynn Ong, Ben K L Ng, Lit Fai Chan, Daphne Koek, Chuen Seng Tan, Falk Müller-Riemenschneider, Karen Cheong, Stijn A A Massar, Michael W L Chee

**Affiliations:** Centre for Sleep and Cognition, Yong Loo Lin School of Medicine, National University of Singapore, 12 Science Drive 2, Singapore 117549, Singapore; Centre for Sleep and Cognition, Yong Loo Lin School of Medicine, National University of Singapore, 12 Science Drive 2, Singapore 117549, Singapore; Health Promotion Board, 3 Second Hospital Ave, Singapore 168937, Singapore; Health Promotion Board, 3 Second Hospital Ave, Singapore 168937, Singapore; Health Promotion Board, 3 Second Hospital Ave, Singapore 168937, Singapore; Saw Swee Hock School of Public Health, National University of Singapore, 12 Science Drive 2, Singapore 117549, Singapore; Saw Swee Hock School of Public Health, National University of Singapore, 12 Science Drive 2, Singapore 117549, Singapore; Berlin Institute of Health, Charite University Medical Centre, Berlin, Germany; Health Promotion Board, 3 Second Hospital Ave, Singapore 168937, Singapore; Centre for Sleep and Cognition, Yong Loo Lin School of Medicine, National University of Singapore, 12 Science Drive 2, Singapore 117549, Singapore; Centre for Sleep and Cognition, Yong Loo Lin School of Medicine, National University of Singapore, 12 Science Drive 2, Singapore 117549, Singapore

**Keywords:** consumer sleep technologies, sleep duration, sleep variability, reliability

## Abstract

**Study Objectives:**

To determine the minimum number of nights required to reliably estimate weekly and monthly mean sleep duration and sleep variability measures from a consumer sleep technology (CST) device (Fitbit).

**Methods:**

Data comprised 107 144 nights from 1041 working adults aged 21–40 years. Intraclass correlation (ICC) analyses were conducted on both weekly and monthly time windows to determine the number of nights required to achieve ICC values of 0.60 and 0.80, corresponding to “good” and “very good” reliability thresholds. These minimum numbers were then validated on data collected 1-month and 1-year later.

**Results:**

Minimally, 3 and 5 nights were required to obtain “good” and “very good” mean weekly total sleep time (TST) estimates, while 5 and 10 nights were required for monthly TST estimates. For weekday-only estimates, 2 and 3 nights were sufficient for weekly time windows while 3 and 7 nights sufficed for monthly time windows. Weekend-only estimates of monthly TST required 3 and 5 nights. TST variability required 5 and 6 nights for weekly time windows, and 11 and 18 nights for monthly time windows. Weekday-only weekly variability required 4 nights for both “good” and “very good” estimates while monthly variability required 9 and 14 nights. Weekend-only estimates of monthly variability required 5 and 7 nights. Error estimates made using data collected 1-month and 1-year later with these parameters were comparable to those associated with the original dataset.

**Conclusions:**

Studies should consider the metric, measurement window of interest, and desired reliability threshold to decide on the minimum number of nights required to assess habitual sleep using CST devices.

**Statement of Significance**

Consumer sleep technologies (CSTs) are increasingly being used for large-scale, longitudinal characterization and improvement of sleep. Sleep duration, timing, and variability are key measures. Using longitudinal data from 1041 office workers collected over 2 years, we formulated robust guidance on the number of nights recommended for estimating weekly and monthly sleep measures at two levels of reliability. More nights are recommended for reliable estimation of monthly compared to weekly time periods, while fewer nights are recommended for sleep timing compared to duration/variability metrics. These findings will assist the development of next-generation sleep guidelines using CSTs and allow researchers to design studies meeting acceptable reliability thresholds for a specific sleep metric and measurement window of interest, with the resources available.

## Introduction

Sleep duration and variability [1, 2] are increasingly recognized as lifestyle factors that can be modified to avert adverse long-term health outcomes [3–7]. The past decade has seen a rapid rise in the adoption of consumer sleep technologies (CSTs) that could facilitate this goal. In 2020 alone, worldwide spending on these devices amounted to $69 billion and is expected to increase [8]. Large-scale, long-term objective sleep tracking via these trackers [9, 10] could improve the assessment of population health, interventions to improve sleep, and realize the dispensation of personalized sleep advice [11–17]. Reliable objective measurement of sleep can also aid in the refinement of sleep recommendations, which are currently based on consensus based on self-report duration, and do not take into account other metrics such as variability.

Earlier concerns about data quality obtained from early consumer sleep trackers are continually being addressed by both improvements in measurement technology and a growing number of rigorous performance evaluation studies demonstrating high correlation (*r*’s > 0.70) of sleep measurements using PSG and/or research actigraphy [18–21], alongside development of a standardized testing framework [22, 23]. This provides increased assurance regarding the reliability of single-night sleep measurements. However, to accurately characterize habitual sleep patterns that influence health outcomes, multiple nights of sleep need to be sampled due to inherent variability in sleep patterns. While sleep trackers are convenient to deploy for extended periods, compliance in wearing the devices varies, especially when longer-term characterization (weeks to months) of sleep behavior is of interest. Trade-offs need to be made between data completeness, resources available to monitor and motivate compliance, and the final number of participants required to answer a specific research question.

In contrast to studies on physical activity [24–33], only a few studies have explored the reliability and accuracy of sleep patterns assessed from longitudinal sleep tracking [34–38]. The latter is important to establish minimal recording duration benchmarks for the accurate estimation of an individual’s sleep patterns. Existing studies on multiple nights of sleep in children/adolescents [34, 35] recommend collecting at least 5 nights of actigraphy for the estimation of habitual sleep parameters such as sleep onset timing, wake after sleep onset, and sleep efficiency while more than 7 nights could be required for estimation of sleep duration. However, most of such studies have used sleep diaries and research-grade actigraphs. Sleep diaries tend to overestimate sleep duration [39] while studies employing research actigraphs are typically short-term and involve relatively small samples [34, 35]. As these studies also do not typically validate their obtained minimum number of nights on data collected from future time periods (1-month or 1-year later), it is unclear how generalizable or stable these estimates are over the longer term. Finally, existing studies do not distinguish between different aggregated periods of sleep (e.g. weekly vs. monthly, weekday-only vs. weekend-only, or consecutive vs. nonconsecutive nights) due to short recording periods (<1 month). Different time windows may be of interest for different studies (e.g. comparing week-to-week changes in sleep patterns following an intervention vs. tracking seasonal changes in sleep or sleep variability over intervals covering vacations compared to regular work weeks).

To address these gaps, we utilized data from a large-scale longitudinal population-health study. Within-subject, objective sleep data from 1951 individuals were collected over the period of 2 years, using a consumer sleep tracker. These data were used to ascertain the minimum number of nights of sleep data needed to establish reliable estimates of sleep duration and variability for weekly and monthly periods. We then verified the robustness of these estimates, by applying the established minimums in a set of holdout future data recorded from the same participants 1-month and 1-year later. These analyses were also repeated for bed and wake time metrics, over weekday-only and weekend-only time periods, as well as over consecutive vs. nonconsecutive nights.

## Methods

### Data source

**Figure 1.**
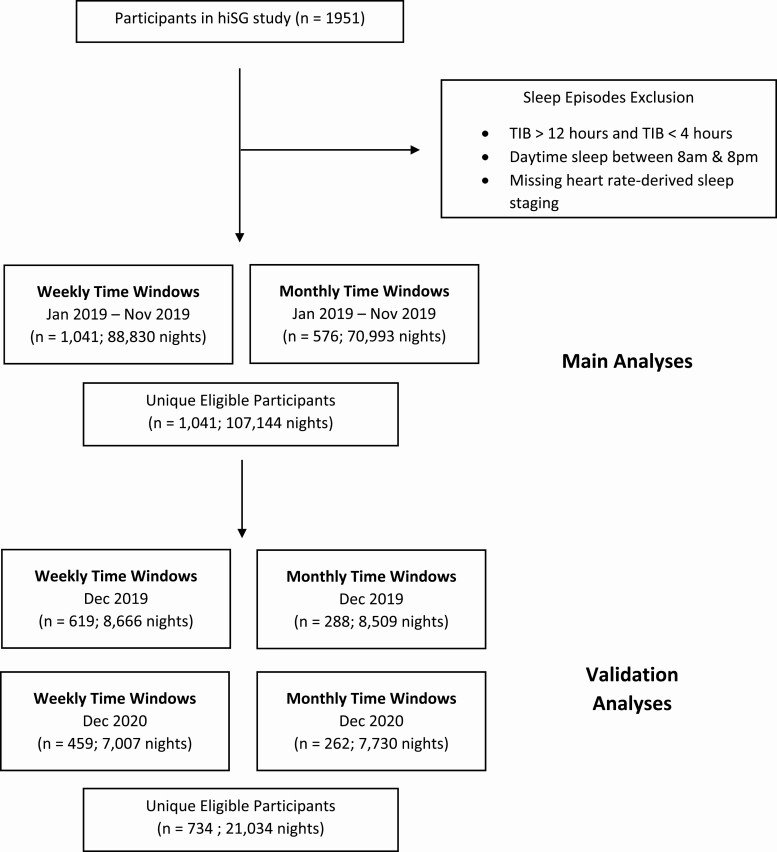
Flow diagram describing inclusion and exclusion criteria of study participants. TIB = Time in Bed. hiSG = Health Insights Singapore study.

Data were obtained from the “Health Insights Singapore” (hiSG) study, a longitudinal population-health study by the Health Promotion Board, Singapore, using wrist-worn sleep trackers paired with a mobile app. Initiated in August 2018, the study recruited 1951 young adults working in the Central Business District aged 21–40 years. As this study intended to survey a representative sample of Singaporean office workers, no sleep exclusion criterion was applied, however, based on a self-report questionnaire probing how well participants thought they slept on a 5-point scale (“Not Well At All,” “Sometimes,” “Neutral,” “Well,” and “Very Well”), 3.8% reported sleeping ‘Not Well At All’ while 13.1% reported only sleeping well ‘Sometimes’. Participants were given devices (Fitbit Ionic, Fitbit Inc, San Francisco, CA) to track their activity/sleep and installed a mobile application to complete surveys over a period of 2 years. They were rewarded with points convertible to vouchers if they wore the tracker daily, logged sleep, meals, and completed surveys and were allowed to keep the device conditional upon meeting study requirements. The National Healthcare Group Domain Specific Review Board approved the study protocol. Informed consent was obtained from all participants prior to study participation. To determine the minimum number of nights for reliably estimating sleep parameters, we utilized data gathered between January 1, 2019 to November 30, 2019 for the main analyses (Figure 1). Two 1-month periods December 2019 and December 2020 were used for validation. Most of the data in 2020 were not used for the main analyses as our earlier work found that lockdowns due to the COVID-19 pandemic affected sleep for this sample [11]. Only participants with full weekly data (7 nights) and 4 weeks of monthly data (28 nights) for each respective weekly and monthly time window were included. In total, we assessed 107 144 nights of sleep data from 1041 participants for the main analyses and 21 034 nights from 734 participants for the validation analyses.

### Tracker based data

Sleep data for each participant were extracted from the Fitbit API (bedtime [startTime], wake time [endTime], time in bed [timeInBed], and total sleep time [minutesAsleep]). Although total sleep time (TST) was the focus of the present manuscript, we also extracted time in bed (TIB), bedtime, and wake time measures in Supplementary Analyses.

Bedtime and wake time were converted to minutes from midnight to simplify analyses. As in our prior work [11], we only analyzed nights where resting heart rate was concurrently collected with sleep data, as this decreased the likelihood of including wrist-off periods where sleep estimation would not be valid. Records that indicated <4 h TIB or >12 h TIB were also excluded from the calculation of sleep variables, as they could indicate possible split sleep sessions or inappropriate detection of sleep by the algorithm (e.g. long periods of sedentary activity after wake). In addition, to exclude atypical sleep periods, we removed sleep sessions that commenced between 08:00 am and 08:00 pm.

### Sampling of sleep data for analyses

Mean and variability estimates for the four sleep parameters: TIB, TST, bedtime, and wake time were assessed. Sleep variability was operationalized as the intraindividual standard deviation of the sampled nights from each time window, defined below.

Since weekly and monthly sleep patterns are important for sleep research and/or assessment of interventions, we analyzed the data across both weekly and monthly time windows. The dataset consisted of 47 weekly (January 1, 2019 to November 30, 2019) and 11 monthly time windows. Monthly time windows were set to a fixed length of 28 nights to ensure consistency and to allow comparisons between different months. For each of these time windows, we separately estimated the minimum number of nights required to reliably estimate sleep parameters. To do this, a variable number of nights were sampled from each time window. The number of nights sampled (referred to as *i* from here) for mean sleep parameters ranged from 1 to 6 nights for weekly time windows and 1 to 27 nights for monthly time windows, while the number of nights for sleep variability parameters ranged from 2 to 6 nights and 2 to 27 nights for weekly and monthly time windows respectively, as variance can only be computed from at least 2 data records. The maximum *i* for each time window was 1 night smaller than the total number of nights in the time window. For example, 6 nights would be the maximum *i* to be drawn for weekly time windows as 7 nights would represent the use of complete data for that week. Next, we applied the sampling method used by Yao et al [29]. For each sample night *i*, 10 sets of samples was drawn without replacement for each participant and the average over the *i* nights was computed. For instance, for a weekly time window (January 1 to January 7), 10 sets of samples of *i* = 6 sample nights were drawn from each participant and averaged over 6 nights, giving 10 sample means of a sleep parameter. This was repeated for *i* = 1 to *i* = 5, for each of the 8 sleep parameters. 10 sets of samples were chosen as our sensitivity analyses showed that drawing extra sets of sizes 20 and 50 did not improve the stability of results.

Additionally, the sampling was performed with two approaches: random nonconsecutive and random consecutive. This was to mirror and account for missing data in both consecutive and nonconsecutive nights in the time series that leads to gaps between nights. For the random nonconsecutive approach, *i* sample nights were sampled without replacement from the specific time window. In the random consecutive approach, a start night of the time window was chosen randomly, and *i* nights were extracted consecutively starting from that point. Time windows were considered as circular time series, i.e. each time window was appended to its end to provide a continuous time window for this period.

To explore the effects of type of day (weekday/weekend) on the minimum number of nights, we examined both combined weekday + weekend data as well as weekday-only (Sun–Thu night) and weekend-only (Fri–Sat night) data. For weekly weekday-only time windows, *i* ranged from 1 to 4 given only 5 weekdays within a week. Weekend-only data were not examined for weekly time windows since *i* would only consist of 1 night which would be insufficient for reliable sleep estimates. For monthly time windows, *i* ranged from 1 to 19 (20 weekdays) and 1 to 7 (8 weekends) for the weekday-only and weekend-only analyses respectively.

### Computation of reliability and error metrics

The metric computed for the main analyses were the intraclass correlation coefficient (ICC). The ICC represents the proportion of between-subject variation over the total variance. The higher this value, the lower the proportion of within-subject variation indicating higher reliability. As the sample means from weekly or monthly data were used to estimate the observed sleep parameters averaged over the same week or month of the complete data, ICC would increase as a function of the number of *i* sample nights. To determine the minimum number of nights to reliably estimate observed weekly and monthly sleep parameters, the smallest *i* sample nights were chosen at the point at which ICC values passed the threshold of 0.6 and 0.8. ICC values in the range of 0.6 to 0.8 indicate “good” reliability and values of above 0.8 indicate “very good” reliability [40]. While researchers should aim to collect enough data to ensure highly reliable estimates, this may not always be possible; here we suggest a lower bound that has “good” levels of reliability.

To determine the error when estimating the observed weekly or monthly sleep parameters of the complete data from a minimum number of nights, we computed the mean absolute error (MAE). However, this alone does not give a representation of its true performance given that the same set of data were used to compute both ICC and the error scores. Thus, we also examined whether our obtained minimum number of nights generalized to a separate dataset, collected on the same individual 1-month and 1-year later. To perform this validation, we computed the MAE for the following month of December 2019, as well as one year later in the month of December 2020. Since these 2 months were not used to compute the ICC, they provide an idea of the generalizability of our results to data independent of the current analyses. The formulae for the MAE for any given *i* sample nights in a time window is as follows:


$$\begin{array}{l} MAE=\;\frac{1}{N}\underset{n=1}{\overset{N}{\sum }}\;\frac{1}{10}\underset{j=1}{\overset{10}{\sum }}\;\;\;(|mean\;daily\;sleep\;from\;i\;sample\;days_{n}-\\ \;mean\;daily\;sleep\;from\;complete\;data_{n}|) \end{array}$$

### Statistical analysis

To compute the ICCs, we used two-way mixed models to obtain estimates of weekly or monthly aggregated metrics (i.e. mean or variability) from the 10 sets of samples [41]. For each time window and *i* sample nights, a mixed model was fitted and the variance components were extracted for ICC computation. This resulted in a set of ICCs for each range of *i* sample nights in any given time window for a sleep parameter (e.g. 6 ICCs for sample nights *i* = 1 to 6 for a random nonconsecutive weekly weekday + weekend combined time window for TST). To obtain an average ICC value for weekly and monthly windows, ICC values for each *i* sample night were averaged across all weeks and all months separately, and the smallest *i* that passed the “good” (ICC ≥ 0.6) and “very good” (ICC ≥ 0.8) [40] threshold were selected, representing the minimum number of nights to estimate weekly and monthly sleep with “good” and “very good” reliability levels respectively.

Nonparametric Friedman tests with Bonferroni corrections were subsequently applied to examine differences between the minimum number of nights obtained from nonconsecutive and consecutive approaches. All analyses were performed in R (version 4.0.5), with mixed models fitted using the ‘lme4’ package (version 1.1.26) and ICCs extracted using the ‘performance’ package (version 0.7.3).

## Results

### Sociodemographic and sleep characteristics

Data from 1041 and 576 participants contributed to analyses of weekly and monthly time windows respectively. Sociodemographic and sleep characteristics of the participants are presented in Table 1. Daily wear time (mean ± standard deviation) averaged 19.40 ± 5.38 h.

**Table 1.** Sociodemographic and sleep summaries (mean and SDs) for eligible participants (n=1041)

*Variable*	Mean (SD) / %
**Sociodemographic features**	
Age	31.15 (4.52)
BMI	23.32 (3.99)
Sex—Females (%)	50.24
Ethnicity—Chinese (%)	94.62
Monthly household earnings in SGD(%)	
<$2k	9.61
$2k–$3.9k	39.39
$4k–$5.9k	26.99
$6k–$7.9k	10.09
$8k–$9.9k	5.76
>=$10k	8.17
Education—Bachelors’/Postgraduate Degree (%)	86.07
**Number of nights of tracking**	
Weekdays and weekends	221.97 (72.68)
Weekdays	162.55 (53.32)
Weekends	59.42 (20.74)
**Sleep**	
Time in bed (min)	428.94 (34.31)
Total sleep time (min)	372.40 (30.15)
Total sleep time variability (min)	63.21 (13.62)
Bedtime (hh:mm)	00:25 (54.22)
Wake time (hh:mm)	07:34 (51.18)
**Daily wear time (h)**	19.40 (5.38)

SGD = Singapore dollars; 1 SGD ~= 0.72 USD.

### Weekly time windows

The minimum number of nights to reliably estimate weekly average sleep was determined using data from 47 weekly time windows and averaging the ICC scores.

**Figure 2.**
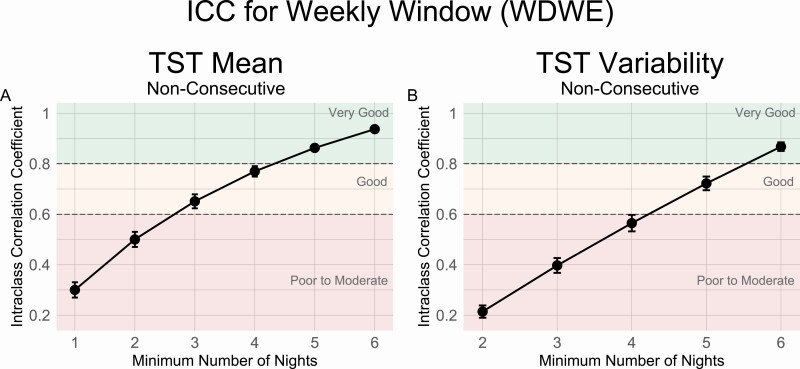
ICC values by the number of sample nights from weekly time windows using nonconsecutive nights for TST (A) Mean and (B) Variability measures. Reliability thresholds of 0.8 and 0.6 are shown in dashed lines.

The ICC analyses suggested that a minimum of 5 nights were needed to obtain a “very good” estimate of weekly average TST (ICC = 0.86, MAE = 11.39 mins), while at least 3 nights were required for a “good” estimate (ICC = 0.65, MAE = 20.84 mins) (Figure 2, A and Table 2). For estimation of weekly TST variability, 6 nights were needed for a “very good” estimate (ICC = 0.87, MAE = 5.62 mins) while 5 nights were needed for a “good” estimate (ICC = 0.72, MAE = 9.75 mins) (Figure 2, B and Table 2). MAEs for the entire range of available nights (1–6) are also presented in the Supplementary (Figure S1).

**Table 2.** Mean absolute errors using minimum number of nights from ICC analyses of weekly time windows

Measure	ICC threshold	Min nights	MAE Jan–Nov 2019	MAE Dec 2019	MAE Dec 2020
**Weekly**					
TST mean (min)	0.8 (very good)	5	11.39 (5.17)	11.85 (5.35)	11.14 (5.23)
	0.6 (good)	3	20.84 (9.56)	21.04 (9.19)	20.46 (9.58)
TST variability (min)	0.8 (very good)	6	5.62 (3.50)	5.75 (3.60)	5.39 (3.44)
	0.6 (good)	5	9.75 (6.05)	9.79 (5.96)	9.37 (5.75)

Means (SDs) are presented for weekly time windows for the minimum number of nights obtained from ICC analyses for 0.8 and 0.6 reliability threshold. Only results for nonconsecutive approaches are displayed here.

The minimum number of nights obtained from the initial analyses were used to estimate complete weekly sleep parameters in two holdout future time windows one month later in December 2019 and one year later in December 2020. Resulting estimates for each threshold had very similar accuracy in the future time periods as were found in the original January–November 2019 time period. (MAE’s differing by <1 min; see Table 2).

Details for other sleep metrics (TIB, bedtime, wake time) are described in the Supplementary Materials. In short, highly similar estimates were found for TIB mean (“good”: 3 nights, ICC = 0.66; “very good”: 5 nights, ICC = 0.87) and variability (“good”: 5 nights, ICC = 0.72; “very good”: 6 nights, ICC = 0.87). For bedtime and wake time, fewer nights were needed for reliable estimates of the mean (bedtime: 2–3 nights; wake time: 2–4 nights) than for variability (bedtime: 5–6 nights; wake time: 5–6 nights; See Supplementary Figure S2 for analysis details for all sleep metrics). Finally, sampling using nonconsecutive and consecutive approaches yielded similar results (*p* = .56), therefore only results for the nonconsecutive approach are presented here (See Supplementary Figure S2 for the consecutive approach and other sleep variables).

### Monthly time windows

The minimum number of nights to reliably estimate complete monthly average sleep was determined using data from 11 monthly time windows. Similar to the weekly time windows, this was determined by taking the smallest number of sample nights that yielded an average ICC that was above the threshold for “good” (ICC ≥ 0.6) and “very good” (ICC ≥ 0.8) levels across the 11 months.

**Figure 3.**
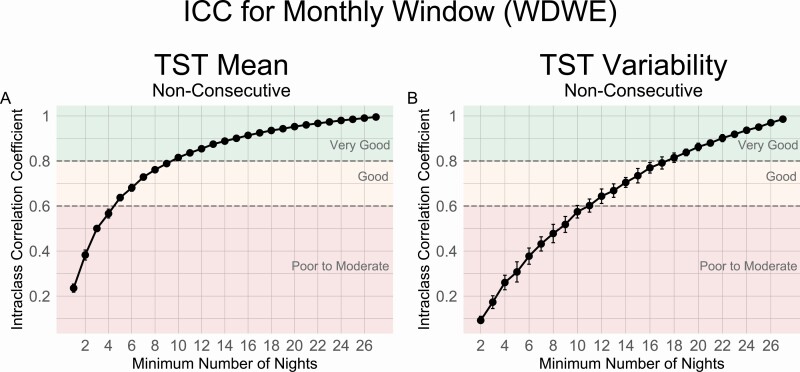
ICC values by the number of sample nights from monthly time windows using nonconsecutive nights for TST (A) Mean and (B) Variability measures. Reliability thresholds of 0.8 and 0.6 are shown in dashed lines.

ICC analyses revealed that a minimum of 10 nights were needed to obtain a “very good” estimate of mean monthly TST (ICC = 0.82, MAE = 11.78 min), while at least 5 nights were required for a “good” estimate of monthly TST (ICC = 0.64, MAE = 18.71 min) (Figure 3, A and Table 3). For estimation of TST variability, 18 nights were required for a “very good” estimate (ICC = 0.81, MAE = 5.49 min), while 11 nights were required for a “good” estimate (ICC = 0.60, MAE = 9.39 min) (Figure 3, B and Table 3). MAEs for the entire range of available nights (1–27) are also presented in (Supplementary Figure S3).

**Table 3.** Mean absolute error using minimum number of nights from ICC analyses of monthly time windows

Measure	ICC threshold	Min nights	MAE Jan–Nov 2019	MAE Dec 2019	MAE Dec 2020
**Monthly**					
TST mean (min)	0.8 (very good)	10	11.78 (4.12)	12.06 (4.10)	11.60 (4.20)
	0.6 (good)	5	18.71 (6.67)	18.52 (6.23)	18.80 (6.60)
TST variability (min)	0.8 (very good)	18	5.49 (2.44)	5.52 (2.40)	5.34 (3.18)
	0.6 (good)	11	9.39 (4.06)	9.07 (3.68)	9.22 (4.80)

Means (SDs) are presented for monthly time windows for the minimum number of nights obtained from ICC analyses for 0.8 and 0.6 reliability threshold. Only results for nonconsecutive approaches are displayed here.

The MAEs for December 2019 and December 2020 (Table 3) demonstrated the robustness of the minimum number of nights recommendations for monthly time windows. Estimates for each threshold had very similar MAEs in the future time periods as in the original January–November 2019 time window (MAE’s differing by <1 min).

Again, results for other sleep metrics (TIB, bedtime, and wake time) and analysis of the consecutive sampling approach are presented in Supplementary Figure S4.

### Weekday-only and weekend-only analyses

The minimum number of nights required to reliably estimate mean TST and variability was also performed on weekday-only and weekend-only periods (Table 4). Specifically, at least 3 and 2 nights were needed to obtain a “very good” and “good” estimate of mean weekly weekday TST respectively while 4 nights were needed to estimate weekly TST variability with “very good” and “good” estimates. For monthly time windows, mean TST on weekdays required at least 7 and 3 days for “very good” and “good” estimates while on weekends, at least 5 and 3 days were needed. Finally, for sleep variability, at least 14 and 9 days were needed for weekday estimates while 7 and 5 days were needed for weekend estimates. Results for other sleep metrics (TIB, bedtime, and wake time) and analysis of the consecutive sampling approach are presented in Supplementary Figures S5–S7.

**Table 4.** Minimum number of nights required for reliable estimation of weekday-only and weekend-only sleep

Measure	Type of day	ICC threshold	Min nights	MAE Jan–Nov 2019
**Weekly**				
TST Mean (min)	Weekday	0.8 (very good)	3	14.17 (8.17)
		0.6 (good)	2	21.35 (12.29)
TST Variability (min)	Weekday	0.8 (very good)	4	7.05 (5.23)
		0.6 (good)	4	7.13 (5.33)
**Monthly**				
TST Mean (min)	Weekday	0.8 (very good)	7	11.82 (4.75)
		0.6 (good)	3	20.40 (8.16)
	Weekend	0.8 (very good)	5	13.27 (5.71)
		0.6 (good)	3	23.03 (9.69)
TST Variability (min)	Weekday	0.8 (very good)	14	5.16 (3.11)
		0.6 (good)	9	9.16 (5.21)
	Weekend	0.8 (very good)	7	4.75 (2.53)
		0.6 (good)	5	11.19 (5.97)

MAEs are presented in Means (SDs).

## Discussion

In this study, we sought to investigate the minimum number of nights required to reliably characterize the average duration and variability of sleep within a given time window (reaching thresholds for “good” [ICC ≥ 0.6] to “very good” [ICC ≥ 0.8] reliability) from a consumer sleep tracker. We found that for a 1-week period, 3–5 nights of sleep data were needed to reliably estimate mean sleep duration (TST), and 5–6 nights of data were required to estimate sleep variability indexed by the standard deviation of TST. To characterize sleep in 1-month window, 5–10 nights and 11–18 nights would be required for mean TST and TST variability respectively. Fewer nights of data might suffice to produce reliable estimates for sleep timing metrics (bedtime, wake time), or when analyzing weekdays only. These minimum requirements apply to data collected 1-month and 1-year later resulting in comparable error estimates to those associated with the original dataset.

The issue of how much data is minimally required—or conversely, how many missing data can be tolerated given resource constraints and imperfect subject compliance—is of vital importance to the design of sleep studies intended to accurately depict habitual sleep patterns and to understand how best to sleep. So far, studies using consumer-grade sleep trackers have applied inclusion criteria on an ad-hoc basis or based on recommendations derived from sleep diaries or actigraphy data. For example, Ong et al [42]. analyzed data from 20k+ Fitbit users to compare sleep duration and timing across multiple countries and age groups. Based on the distribution of data provided per user, they set a minimum cutoff of 10 weekday nights and 4 weekend nights for inclusion. Another study examined sleep data from over 150k Fitbit users to assess changes in sleep over the 2019 and 2020 Covid-19 pandemic [16]. Following the SBSM guidelines for actigraphy monitoring, a 10-day minimum criterion was applied to calculate habitual sleep duration and timing [43]. Different cutoffs have also been applied in other consumer-grade sleep tracker studies (e.g. 70% of nights within a period [44], 7 consecutive days [45], 14 days [46], and 4 weeks [47]). With the current analysis we aimed to provide a set of recommendations that could be used to allow a more uniform and principled application of minimum data requirements in wearable-based sleep studies for quality control and optimal data selection. This may be particularly pertinent for large-scale and/or longitudinal sleep tracking studies, as missing data can be expected to scale with increased tracking time [48], or when analyzing legacy datasets (e.g. from open sources or when end-user data is analyzed through the device manufacturers’ databases [16]). As our supplementary analyses reveal large MAEs arising from the use of too few nights of data, it is crucial that reliable sleep estimates are obtained from sufficient nights in order that correct estimates for sleep recommendations can be derived.

A notable strength of the current analysis was that it was based on a large-scale longitudinal dataset. Data from 1041 individuals over the course of 11 months were included in the initial analysis. This allowed us to arrive at robust reliability estimates. Furthermore, the longitudinal nature of the dataset allowed us to validate the applicability of the found minimum data recommendations against future time periods (as far as one year later). Using the minimum requirements as derived from the initial 11 months yielded very similar reliability estimates when applied on data collected 1 month and 1 year later. This further affirms the stability and utility of these estimates for the characterization of longer-term habitual sleep patterns. These features set the current study apart from previous actigraphy/sleep diary-based studies that were mostly based on data collection over shorter periods of time (one to several weeks [34–37]) and render the resulting recommendations particularly applicable for longitudinal data. While these conclusions could conceivably be applied on multiday studies using research-grade accelerometers, our findings of 3–5 nights for reliable estimation of weekday/weekday–weekend combined sleep duration did differ from prior work in adults suggesting that more than 7 nights would be required [35]. However, as analysis methods used in these studies differed, it is still unclear whether similar conclusions would be obtained with research-grade accelerometers.

Another advantage of the current data were that it allowed us to calculate reliability estimates over different time windows. Monthly time windows may be of particular interest in population studies where aggregated data can be used to assess long-term trends in sleep patterns (e.g. examining the effects of evolving safety measures during the COVID-19 pandemic [11, 16]). Other studies, such as sleep intervention studies, may rely more on assessing changes in sleep over shorter time periods as short as one week (e.g. comparing baseline versus post-intervention sleep [12, 49, 50]). One finding of our analysis was that while more nights of data were required to reliably characterize a 1-month period compared to a 1-week period, proportionally, a 1-month period is more robust to missing data (requiring 17%–30% of nights for mean TST) than a 1-week period (requiring 43%–60% of nights for mean TST). Supplementary analyses also showed that it did not matter if these nights were consecutively measured or not, allowing for temporal gaps in the data, which often arise due to battery issues, or forgetting to wear the device after charging.

Finally, the data allowed us to separately estimate the minimum data requirements to characterize sleep on weekdays and weekends. Weekend–weekday differences contribute to intraweek variability, and lower reliability estimates. For this reason, some studies focus on weekday sleep only [49]. Our results showed that fewer nights of data were required when weekday sleep was considered in isolation. However, if monthly weekend–weekday differences are of specific interest, it is recommended to include at least 3–7 weekdays and 3–5 weekend nights for mean sleep duration and 9–14 weekdays/5–7 weekend nights for TST variability.

### Limitations and future directions

There are several limitations to note for the current study. First, the dataset was based on a relatively homogenous population of young adult white-collar workers. This precluded a more detailed analysis of sociodemographic factors and the influence that they may have on the reliability estimates. It is likely that examining a wider range of age (e.g. children/adolescents, elderly), occupation (e.g. shift workers, gig workers), and socioeconomic status would lead to more diverse sleep patterns that would consequently influence the reliability estimates. Future studies should verify the current minimum requirements in different populations. Furthermore, validation of the current findings in different geographical locations would be recommended. Seasonal changes in sleeping patterns will likely affect the reliability estimates. As the data were collected in Singapore (located at equatorial latitude), seasonal variation was not present. It is possible that some variation could be introduced due to international travel (not explicitly identified in this study), which could be random across the sample, or most influential during major holiday periods. Finally, future work utilizing long-term monitoring (>14 nights) could also examine minimal requirements for other sleep behaviors e.g. daytime sleep, shorter sleep periods <4 h, sleep fragmentation, or sleep extension before a period of sleep restriction, as this could have an impact on subsequent sleep and performance [51].

## Conclusion

Findings from the current study suggest that average short-term weekly and longer-term monthly sleep from a consumer sleep tracker can be reliably estimated from data with limited or missing data. While obtaining complete data for sleep duration, timing, and variability are ideally preferred, this is not always possible in longitudinal designs or consumer-based data analysis. Researchers should plan for an appropriate minimum data collection period, given the sleep measure and time frame of interest. We show that to achieve reliable estimates of mean sleep duration over a 1-week period, a minimum of 3–5 nights would be required for mean sleep duration and 5–6 nights for a 1-month period. Variability measures required more nights than mean estimates, and sleep timing measures required fewer nights than sleep duration estimates. Since these measures are usually derived concurrently from consumer sleep trackers, collecting 6 nights (for 1-week) or 19 nights (for 1-month) of data should reasonably cover the most commonly tracked sleep measures.

## Supplementary Material

zpac026_suppl_Supplementary_Material

## Data Availability

Aggregate data will be made available upon reasonable request.
