# Supplementary material for: Minimum number of nights for reliable estimation of habitual sleep using a consumer sleep tracker
Source: Sleep Adv. 2022 Aug 31;3(1):zpac026. doi: 10.1093/sleepadvances/zpac026 (PMC10104388; doi:10.1093/sleepadvances/zpac026)
Supplement: zpac026_suppl_Supplementary_Material [file zpac026_suppl_supplementary_material.docx]

**Minimum number of nights for reliable estimation of habitual sleep using a consumer sleep tracker**

TeYang Lau^1^, Ju Lynn Ong^1^, Ben K L Ng^3^, Lit Fai Chan^3^, Daphne Koek^3^, Chuen Seng Tan^2^, Falk Müller‑Riemenschneider^2,4^, Karen Cheong^3^, Stijn A A Massar^1^, Michael W L Chee^1^*

^1^Centre for Sleep and Cognition, Yong Loo Lin School of Medicine, National University of Singapore, 12 Science Drive 2, Singapore 117549, Singapore.

^2^Saw Swee Hock School of Public Health, National University of Singapore, 12 Science Drive 2, Singapore 117549, Singapore.

^3^Health Promotion Board, 3 Second Hospital Ave, Singapore 168937, Singapore.

^4^Berlin Institute of Health, Charite University Medical Centre, Berlin, Germany.

* Corresponding author:

Michael W.L. Chee

Professor and Director,

Center for Sleep and Cognition,

Yong Loo Lin School of Medicine,

12 Science Drive 2,

National University of Singapore,

Singapore 117549.

Email: [michael.chee@nus.edu.sg](mailto:michael.chee@nus.edu.sg)

**Supplementary Materials**

**Analysis of non-consecutive vs consecutive approaches**

**Weekly Time Windows**

Results for minimum number of days were similar for both random non-consecutive and random consecutive approaches (Figure S2), with only the non-consecutive approach (mean=3.85, SD=0.36) requiring slightly fewer days compared to the consecutive approach (mean=4.13, SD=0.45) for waketime (*p* = .002).

For the weekday-only analysis of sleep mean, TIB and TST required 3 days while bedtime and wake time required 2 days (Figure S5A & S5C) for “very good” estimates regardless of the continuity of the sampled days (all *p*s > .05). For sleep variability, all sleep variables required 4 days for “very good” estimates (Figure S5B & 52D).

Mean absolute errors (MAEs) using these minimum number of days are presented in Table S1 for Jan-Nov 2019, Table S2 for Dec 2019, and Table S3 for Dec 2020.

**Monthly Time Windows**

Results for minimum number of days were similar for both random non-consecutive and random consecutive approaches (Figure S4) (all *p*s > .08).

For weekday-only analysis within a month, TIB and TST required 6 and 7 days respectively for non-consecutive days (Figure S6A) while both TIB and TST needed 7 days for consecutive days (Figure S6C), but this difference was not significant (all *p*s > .03). On the other hand, only 4 days were required to reliably estimate bed/wake timings for both consecutive and non-consecutive days (all *p*s > .03, Figure S46 & S6C).

Finally, 5 and 4 days were needed to reliably estimate non-consecutive and consecutive weekend TIB and TST respectively, while both bedtime and waketime required 3 days regardless of the continuity of days (all *p*s > .16*,* Figure S7A & S7C).

MAEs using these minimum number of days are presented in Table S1 for Jan-Nov 2019, Table S2 for Dec 2019, and Table S3 for Dec 2020.


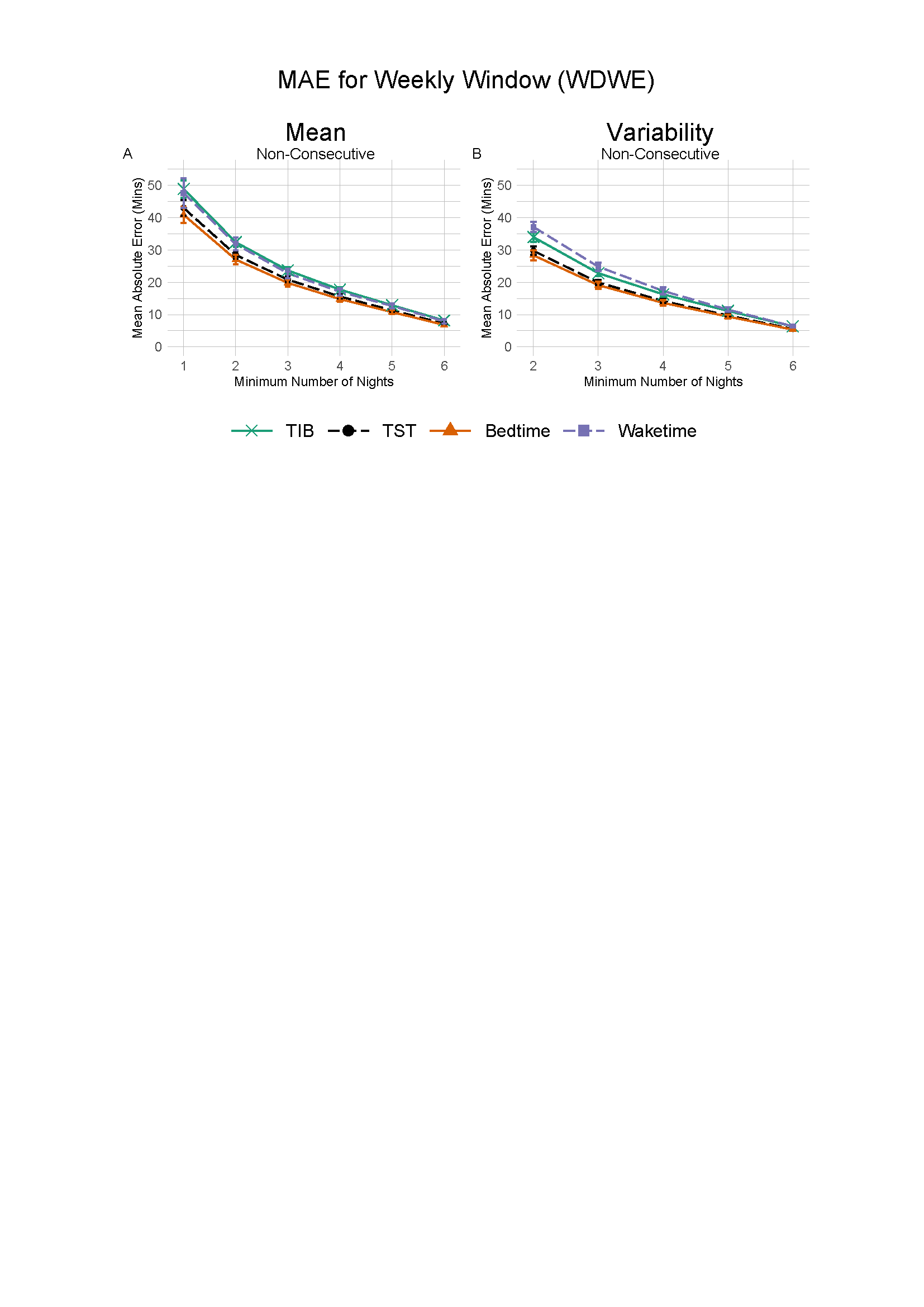


Figure S1. MAEs for each number of sample days for **weekly weekday-weekend** combined time windows. A) Sleep mean variables using non-consecutive days. B) Sleep variability variables using non-consecutive days.


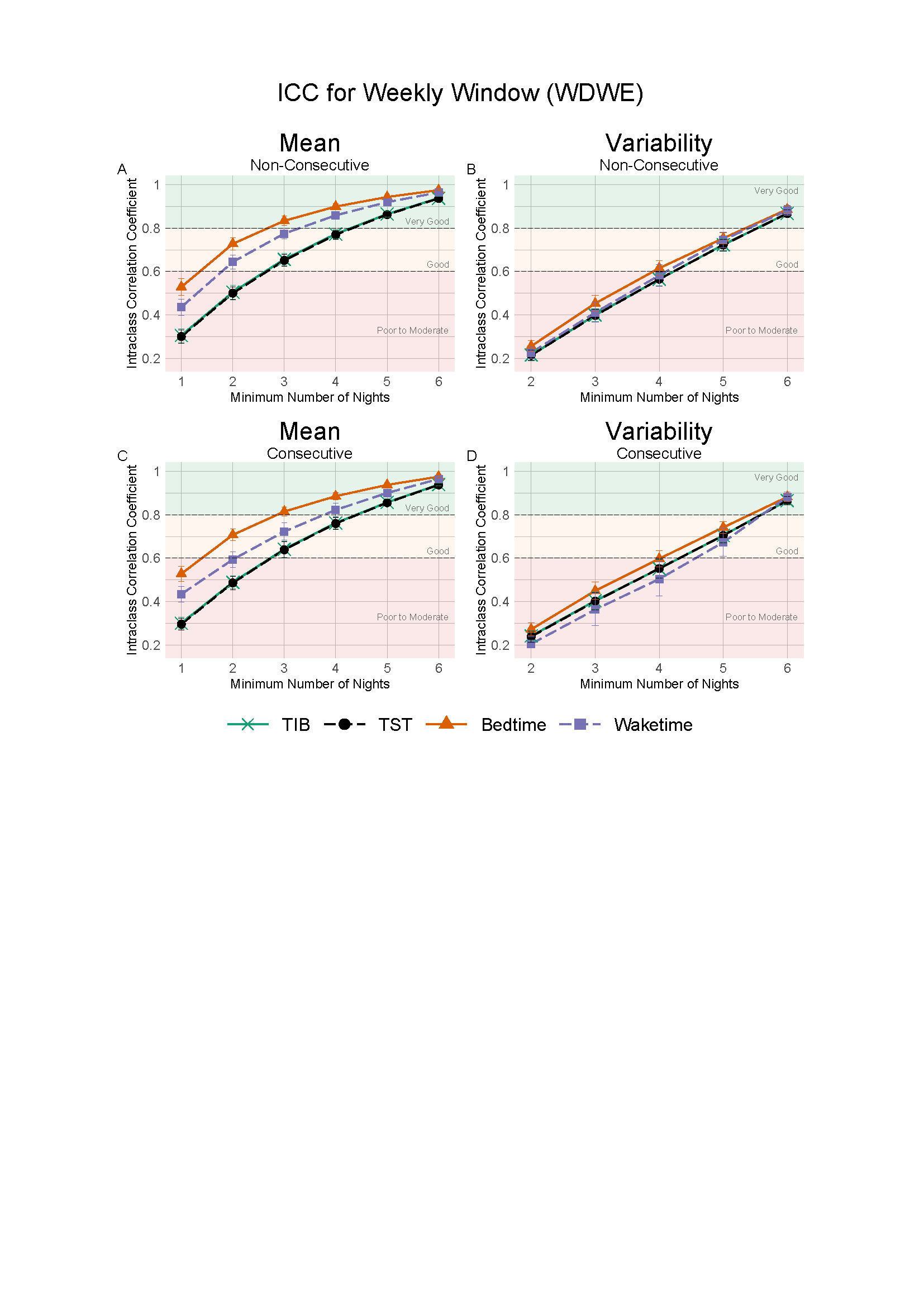


Figure S2. ICCs for each number of sample days for **weekly weekday-weekend** combined time windows. Reliability thresholds of 0.6 and 0.8 are shown in dashed lines. A) Sleep mean variables using non-consecutive days. B) Sleep variability variables using non-consecutive days. C) Sleep mean variables using consecutive days. D) Sleep variability variables using consecutive days.


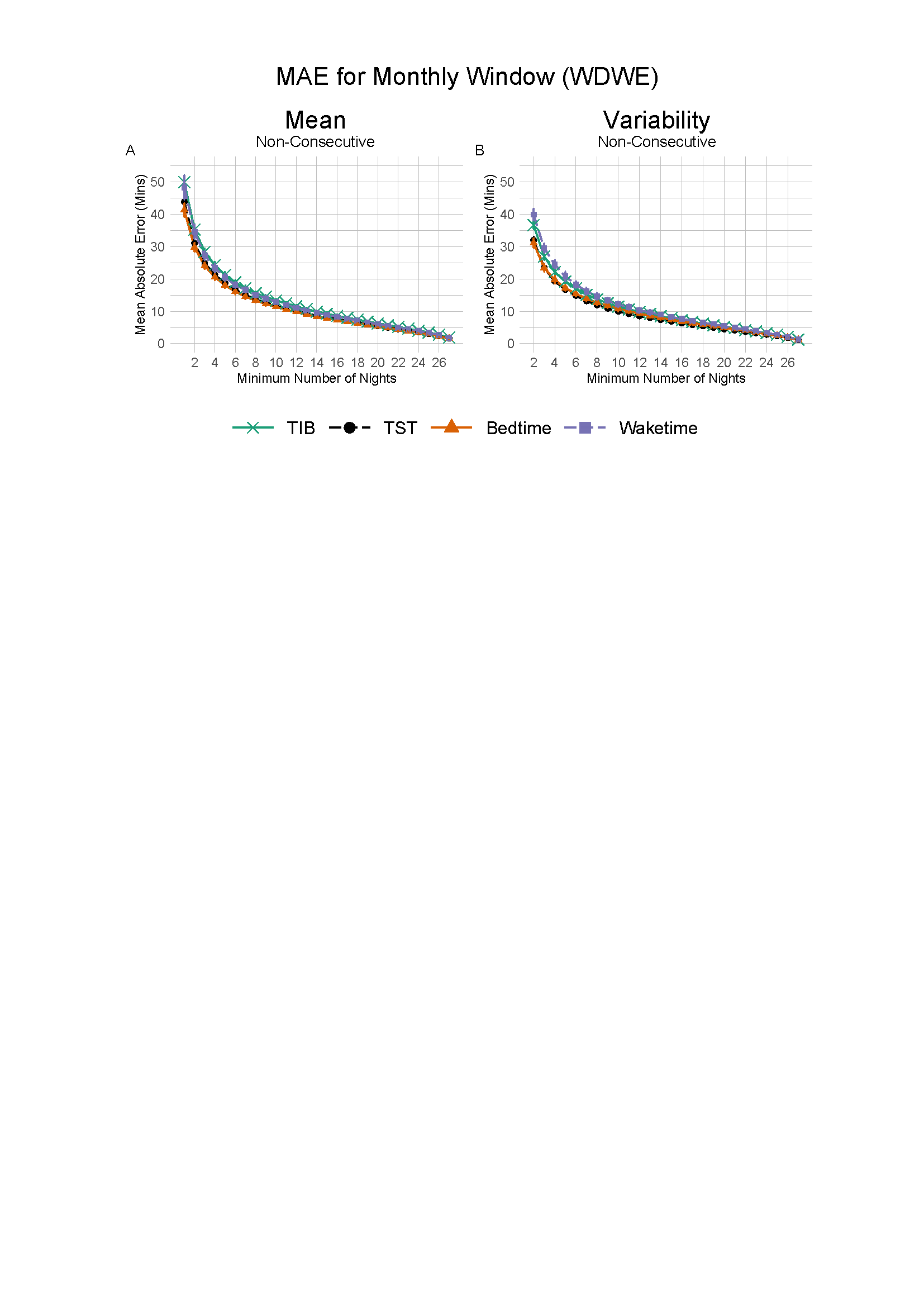


Figure S3. MAEs for each number of sample days for **monthly weekday-weekend** combined time windows. A) Sleep mean variables using non-consecutive days. B) Sleep variability variables using non-consecutive days.


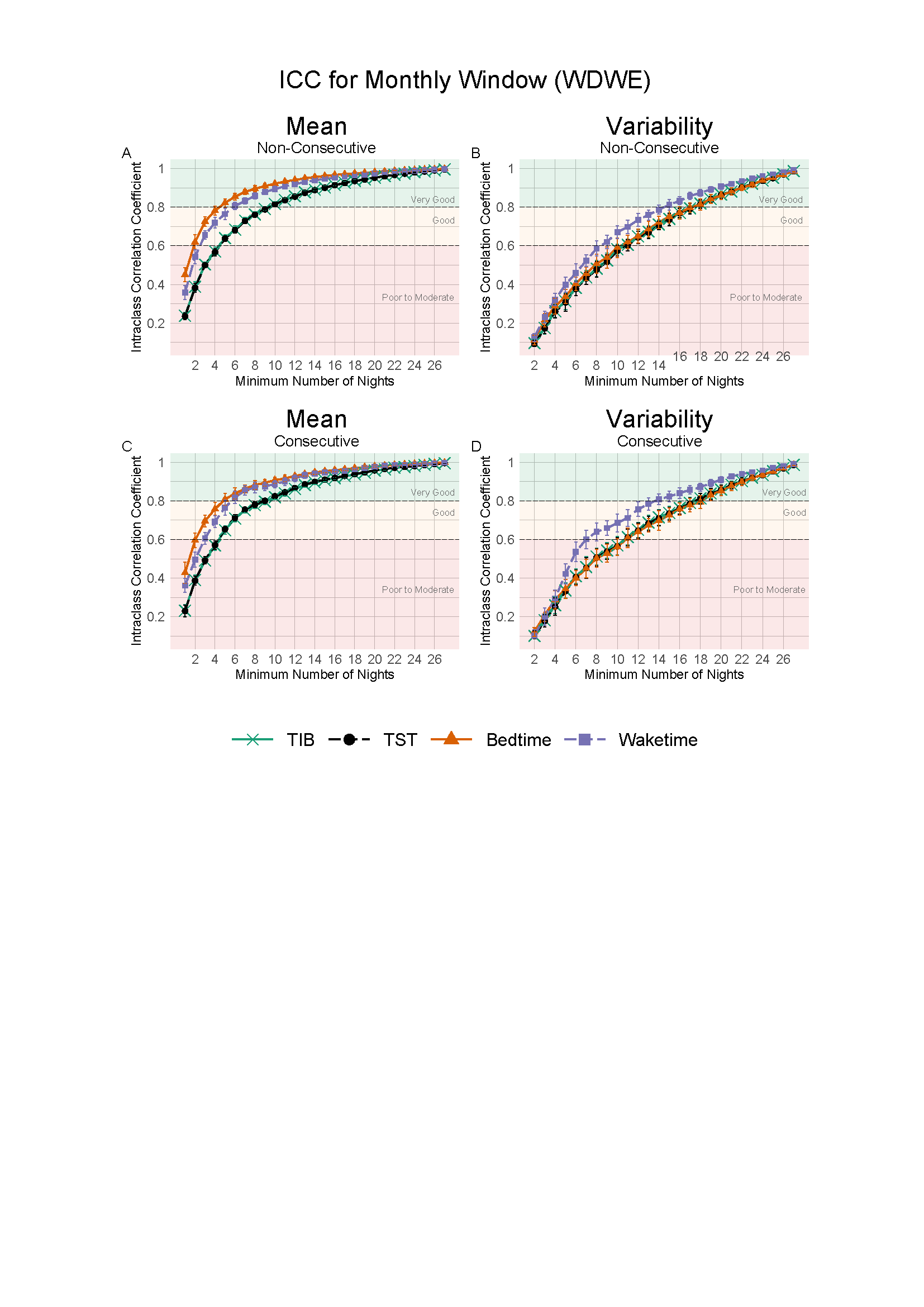


Figure S4. ICCs for each number of sample days for **monthly weekday-weekend** combined time windows. Reliability thresholds of 0.6 and 0.8 are shown in dashed lines. A) Sleep mean variables using non-consecutive days. B) Sleep variability variables using non-consecutive days. C) Sleep mean variables using consecutive days. D) Sleep variability variables using consecutive days.


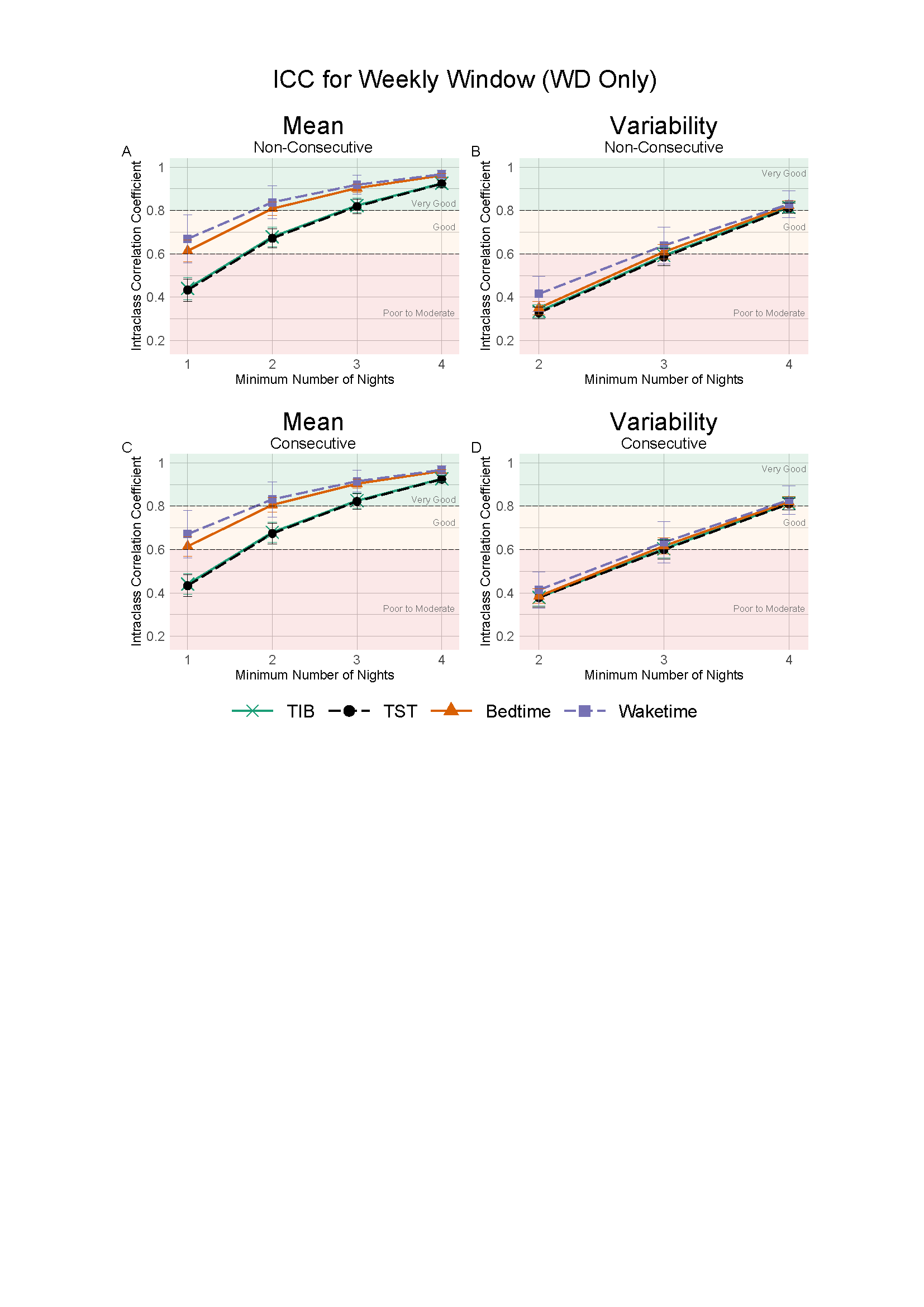


Figure S5. ICCs for each number of sample days for **weekly weekday** time windows. Reliability thresholds of 0.6 and 0.8 are shown in dashed lines. A) Sleep mean variables using non-consecutive days. B) Sleep variability variables using non-consecutive days. C) Sleep mean variables using consecutive days. D) Sleep variability variables using consecutive days.


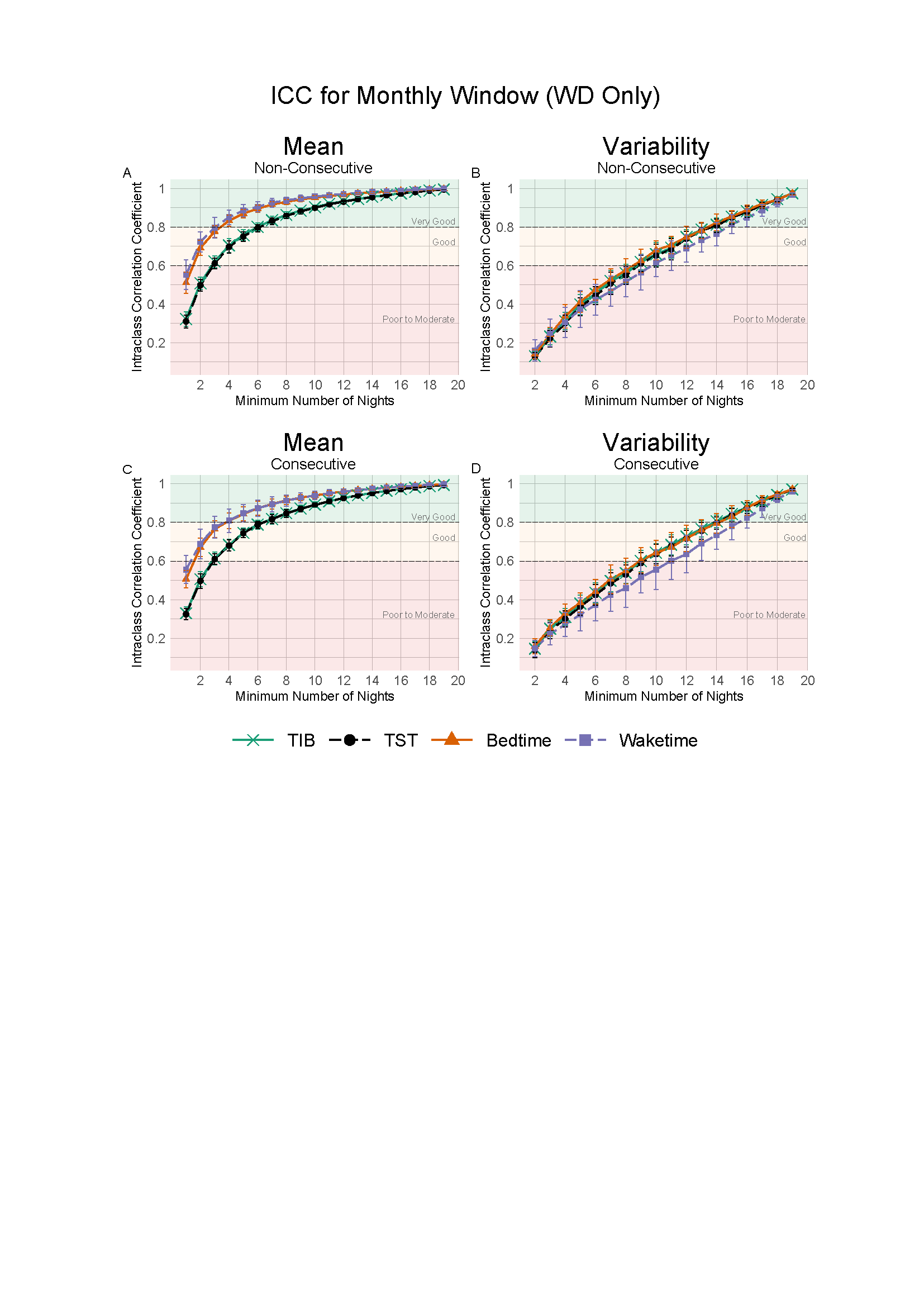


Figure S6. ICCs for each number of sample days for **monthly weekday** time windows. Reliability thresholds of 0.6 and 0.8 are shown in dashed lines. A) Sleep mean variables using non-consecutive days. B) Sleep variability variables using non-consecutive days. C) Sleep mean variables using consecutive days. D) Sleep variability variables using consecutive days.


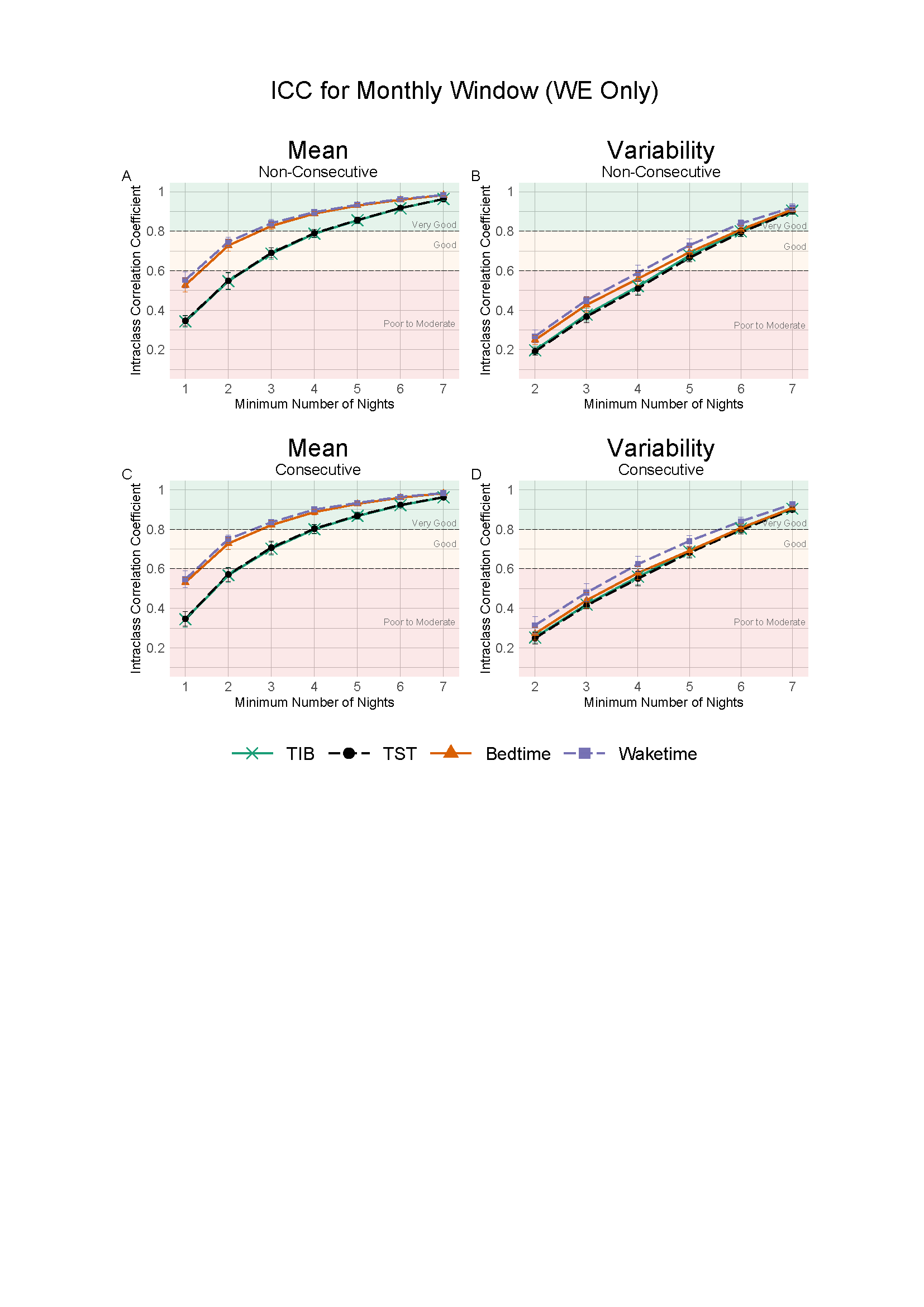


Figure S7. ICCs for each number of sample days for **monthly weekend** time windows. Reliability thresholds of 0.6 and 0.8 are shown in dashed lines. A) Sleep mean variables using non-consecutive days. B) Sleep variability variables using non-consecutive days. C) Sleep mean variables using consecutive days. D) Sleep variability variables using consecutive days.

Table S1. Mean Absolute Error for Jan-Nov 2019 Using Minimum Number of Days from ICC Analyses of Sleep Mean and Sleep Variability

| Variable | Sampling | Threshold | Min Days | Time In Bed | Min Days | Total Sleep Time | Min Days | Bedtime | Min Days | Waketime |
| --- | --- | --- | --- | --- | --- | --- | --- | --- | --- | --- |
| **Weekly** |  |  |  |  |  |  |  |  |  |  |
| Weekday  Sleep Mean | Non-consec | 0.8 | 3 | 16.02 (9.40) | 3 | 14.17 (8.17) | 2 | 21.36 (13.48) | 2 | 16.52 (15.89) |
|  | Non-consec | 0.6 | 2 | 24.09 (14.11) | 2 | 21.35 (12.29) | 1 | 34.23 (21.56) | 1 | 25.51 (25.03) |
|  | Consec | 0.8 | 3 | 15.99 (10.28) | 3 | 14.11 (8.92) | 2 | 21.51 (14.69) | 2 | 16.94 (16.96) |
|  | Consec | 0.6 | 2 | 23.99 (15.40) | 2 | 21.16 (13.33) | 1 | 34.08 (21.25) | 1 | 25.47 (24.71) |
|  |  |  |  |  |  |  |  |  |  |  |
| Weekday  Sleep Variability | Non-consec | 0.8 | 4 | 8.04 (6.07) | 4 | 7.05 (5.23) | 4 | 7.17 (5.84) | 4 | 6.29 (7.50) |
|  | Non-consec | 0.6 | 4 | 8.00 (6.00) | 4 | 7.13 (5.33) | 4 | 13.43 (10.63) | 3 | 12.05 (13.79) |
|  | Consec | 0.8 | 4 | 8.05 (6.08) | 4 | 7.11 (5.32) | 4 | 7.21 (5.99) | 4 | 6.32 (7.63) |
|  | Consec | 0.6 | 3 | 15.06 (11.31) | 3 | 13.23 (9.68) | 3 | 13.48 (10.98) | 3 | 12.39 (14.36) |
|  |  |  |  |  |  |  |  |  |  |  |
| **Monthly** |  |  |  |  |  |  |  |  |  |  |
| Weekday  Sleep Mean | Non-consec | 0.8 | 6 | 15.09 (6.16) | 7 | 11.82 (4.75) | 4 | 17.6 (7.91) | 4 | 15.11 (9.01) |
|  | Non-consec | 0.6 | 3 | 23.27 (9.45) | 3 | 20.4 (8.16) | 2 | 26.02 (11.53) | 2 | 21.18 (13.31) |
|  | Consec | 0.8 | 7 | 14.04 (7.57) | 7 | 12.15 (6.51) | 4 | 18.67 (9.51) | 4 | 16.93 (11.50) |
|  | Consec | 0.6 | 3 | 23.71 (10.95) | 3 | 20.75 (9.39) | 2 | 26.43 (12.41) | 2 | 22.07 (14.73) |
|  |  |  |  |  |  |  |  |  |  |  |
| Weekend  Sleep Mean | Non-consec | 0.8 | 5 | 15.22 (6.78) | 5 | 13.27 (5.71) | 3 | 21.38 (11.06) | 3 | 22.74 (12.20) |
|  | Non-consec | 0.6 | 3 | 26.27 (11.18) | 3 | 23.03 (9.69) | 2 | 28.48 (14.04) | 2 | 30.35 (16.27) |
|  | Consec | 0.8 | 4 | 20.42 (11.69) | 4 | 17.68 (9.87) | 3 | 22.66 (13.11) | 3 | 24.07 (14.95) |
|  | Consec | 0.6 | 3 | 26.69 (14.25) | 3 | 23.12 (12.01) | 2 | 29.77 (16.70) | 2 | 30.95 (18.40) |
|  |  |  |  |  |  |  |  |  |  |  |
| Weekday  Sleep Variability | Non-consec | 0.8 | 14 | 5.89 (3.38) | 14 | 5.16 (3.11) | 14 | 5.42 (3.77) | 15 | 6.09 (4.75) |
|  | Non-consec | 0.6 | 9 | 10.41 (5.91) | 9 | 9.16 (5.21) | 9 | 9.61 (6.70) | 10 | 11.48 (8.81) |
|  | Consec | 0.8 | 14 | 6.21 (3.79) | 15 | 4.68 (2.77) | 15 | 4.97 (3.60) | 16 | 5.38 (4.17) |
|  | Consec | 0.6 | 10 | 9.90 (6.27) | 10 | 8.70 (5.63) | 9 | 10.25 (7.77) | 11 | 11.38 (9.16) |
|  |  |  |  |  |  |  |  |  |  |  |
| Weekend  Sleep Variability | Non-consec | 0.8 | 7 | 5.41 (2.93) | 7 | 4.75 (2.53) | 6 | 7.87 (5.45) | 6 | 8.20 (5.59) |
|  | Non-consec | 0.6 | 5 | 12.76 (6.73) | 5 | 11.19 (5.97) | 5 | 11.12 (7.71) | 5 | 11.69 (8.25) |
|  | Consec | 0.8 | 6 | 9.09 (5.18) | 6 | 7.96 (4.54) | 6 | 7.86 (5.51) | 6 | 8.14 (5.89) |
|  | Consec | 0.6 | 5 | 12.76 (7.73) | 5 | 11.11 (6.62) | 4 | 15.05 (10.78) | 4 | 15.63 (11.11) |
|  |  |  |  |  |  |  |  |  |  |  |

Means (SDs) are presented for weekly and monthly time windows for the minimum number of days obtained from ICC analyses for 0.8 and 0.6 reliability threshold. Results are partitioned into weekday only and weekend only analyses.

Table S2. Mean Absolute Error for Dec 2019 Using Minimum Number of Days from ICC Analyses of Sleep Mean and Sleep Variability

| Variable | Sampling | Threshold | Min Days | Time In Bed | Min Days | Total Sleep Time | Min Days | Bedtime | Min Days | Waketime |
| --- | --- | --- | --- | --- | --- | --- | --- | --- | --- | --- |
| **Weekly** |  |  |  |  |  |  |  |  |  |  |
| Weekday  Sleep Mean | Non-consec | 0.8 | 3 | 17.34 (10.05) | 3 | 15.22 (8.69) | 2 | 22.40 (14.06) | 2 | 19.12 (16.81) |
|  | Non-consec | 0.6 | 2 | 25.66 (14.60) | 2 | 22.67 (12.73) | 1 | 35.58 (21.78) | 1 | 29.97 (26.37) |
|  | Consec | 0.8 | 3 | 17.27 (10.84) | 3 | 15.34 (9.52) | 2 | 22.36 (15.65) | 2 | 19.69 (18.19) |
|  | Consec | 0.6 | 2 | 26.23 (16.73) | 2 | 22.83 (14.41) | 1 | 35.11 (21.10) | 1 | 29.57 (26.03) |
|  |  |  |  |  |  |  |  |  |  |  |
| Weekday  Sleep Variability | Non-consec | 0.8 | 4 | 8.44 (6.36) | 4 | 7.55 (5.68) | 4 | 7.45 (5.85) | 4 | 7.01 (8.03) |
|  | Non-consec | 0.6 | 4 | 8.50 (6.22) | 4 | 7.50 (5.86) | 4 | 13.77 (10.60) | 3 | 13.59 (14.25) |
|  | Consec | 0.8 | 4 | 8.50 (6.46) | 4 | 7.46 (5.59) | 4 | 7.36 (5.70) | 4 | 7.07 (7.81) |
|  | Consec | 0.6 | 3 | 15.89 (11.65) | 3 | 14.07 (10.29) | 3 | 13.96 (10.86) | 3 | 13.69 (14.16) |
|  |  |  |  |  |  |  |  |  |  |  |
| **Monthly** |  |  |  |  |  |  |  |  |  |  |
| Weekday  Sleep Mean | Non-consec | 0.8 | 6 | 16.72 (6.86) | 7 | 12.58 (4.53) | 4 | 19.51 (8.35) | 4 | 17.64 (9.97) |
|  | Non-consec | 0.6 | 3 | 25.49 (10.46) | 3 | 22.55 (8.55) | 2 | 28.36 (12.84) | 2 | 25.50 (14.54) |
|  | Consec | 0.8 | 7 | 15.31 (7.46) | 7 | 13.29 (6.59) | 4 | 21.50 (11.40) | 4 | 20.18 (13.87) |
|  | Consec | 0.6 | 3 | 26.23 (11.09) | 3 | 22.23 (9.49) | 2 | 30.04 (13.73) | 2 | 27.26 (16.76) |
|  |  |  |  |  |  |  |  |  |  |  |
| Weekend  Sleep Mean | Non-consec | 0.8 | 5 | 15.62 (6.74) | 5 | 14.02 (5.28) | 3 | 23.41 (12.29) | 3 | 25.68 (13.73) |
|  | Non-consec | 0.6 | 3 | 27.32 (12.22) | 3 | 23.39 (9.66) | 2 | 31.08 (15.88) | 2 | 33.04 (17.54) |
|  | Consec | 0.8 | 4 | 21.00 (11.46) | 4 | 18.14 (9.35) | 3 | 24.34 (15.83) | 3 | 26.26 (16.98) |
|  | Consec | 0.6 | 3 | 26.89 (13.10) | 3 | 22.91 (10.82) | 2 | 31.17 (18.01) | 2 | 34.08 (20.06) |
|  |  |  |  |  |  |  |  |  |  |  |
| Weekday  Sleep Variability | Non-consec | 0.8 | 14 | 6.07 (3.42) | 14 | 5.26 (2.60) | 14 | 5.72 (3.28) | 15 | 5.88 (3.93) |
|  | Non-consec | 0.6 | 9 | 11.01 (5.67) | 9 | 9.24 (4.81) | 9 | 9.97 (5.38) | 10 | 10.92 (7.49) |
|  | Consec | 0.8 | 14 | 6.39 (3.89) | 15 | 4.82 (2.66) | 15 | 5.31 (3.57) | 16 | 5.42 (3.55) |
|  | Consec | 0.6 | 10 | 10.30 (6.64) | 10 | 8.51 (5.29) | 9 | 10.83 (7.42) | 11 | 11.52 (8.61) |
|  |  |  |  |  |  |  |  |  |  |  |
| Weekend  Sleep Variability | Non-consec | 0.8 | 7 | 5.29 (2.87) | 7 | 4.89 (2.44) | 6 | 8.33 (5.99) | 6 | 8.89 (6.17) |
|  | Non-consec | 0.6 | 5 | 12.99 (6.66) | 5 | 11.55 (5.30) | 5 | 12.17 (8.15) | 5 | 12.44 (8.60) |
|  | Consec | 0.8 | 6 | 9.10 (5.09) | 6 | 8.19 (4.25) | 6 | 8.55 (5.91) | 6 | 9.02 (6.60) |
|  | Consec | 0.6 | 5 | 13.31 (7.64) | 5 | 11.37 (6.14) | 4 | 16.68 (11.75) | 4 | 16.87 (11.95) |
|  |  |  |  |  |  |  |  |  |  |  |

Means (SDs) are presented for weekly and monthly time windows for the minimum number of days obtained from ICC analyses for 0.8 and 0.6 reliability threshold. Results are partitioned into weekday-only and weekend-only analyses.

Table S3. Mean Absolute Error for Dec 2020 Using Minimum Number of Days from ICC Analyses of Sleep Mean and Sleep Variability

| Variable | Sampling | Threshold | Min Days | Time In Bed | Min Days | Total Sleep Time | Min Days | Bedtime | Min Days | Waketime |
| --- | --- | --- | --- | --- | --- | --- | --- | --- | --- | --- |
| **Weekly** |  |  |  |  |  |  |  |  |  |  |
| Weekday  Sleep Mean | Non-consec | 0.8 | 3 | 17.49 (10.05) | 3 | 15.48 (9.05) | 2 | 22.43 (14.17) | 2 | 20.41 (15.14) |
|  | Non-consec | 0.6 | 2 | 26.31 (14.89) | 2 | 23.41 (13.76) | 1 | 35.63 (23.59) | 1 | 31.61 (23.64) |
|  | Consec | 0.8 | 3 | 17.19 (10.58) | 3 | 15.37 (9.88) | 2 | 22.73 (15.77) | 2 | 20.28 (15.78) |
|  | Consec | 0.6 | 2 | 25.85 (16.08) | 2 | 23.09 (14.52) | 1 | 36.02 (23.25) | 1 | 32.25 (24.80) |
|  |  |  |  |  |  |  |  |  |  |  |
| Weekday  Sleep Variability | Non-consec | 0.8 | 4 | 8.62 (5.91) | 4 | 7.60 (5.40) | 4 | 7.69 (6.17) | 4 | 7.05 (6.61) |
|  | Non-consec | 0.6 | 4 | 8.72 (6.14) | 4 | 7.71 (5.71) | 4 | 14.26 (10.78) | 3 | 13.27 (11.64) |
|  | Consec | 0.8 | 4 | 8.53 (5.82) | 4 | 7.65 (5.54) | 4 | 7.63 (6.32) | 4 | 6.95 (6.40) |
|  | Consec | 0.6 | 3 | 15.69 (10.88) | 3 | 14.19 (10.21) | 3 | 14.23 (11.21) | 3 | 13.33 (12.12) |
|  |  |  |  |  |  |  |  |  |  |  |
| **Monthly** |  |  |  |  |  |  |  |  |  |  |
| Weekday  Sleep Mean | Non-consec | 0.8 | 6 | 16.47 (6.11) | 7 | 12.84 (5.50) | 4 | 18.08 (7.78) | 4 | 17.35 (9.33) |
|  | Non-consec | 0.6 | 3 | 25.65 (10.72) | 3 | 22.08 (8.33) | 2 | 26.95 (11.68) | 2 | 25.24 (13.39) |
|  | Consec | 0.8 | 7 | 14.93 (7.48) | 7 | 12.81 (7.15) | 4 | 19.60 (9.59) | 4 | 19.51 (11.27) |
|  | Consec | 0.6 | 3 | 25.48 (11.37) | 3 | 21.96 (10.40) | 2 | 28.22 (13.18) | 2 | 26.37 (15.42) |
|  |  |  |  |  |  |  |  |  |  |  |
| Weekend  Sleep Mean | Non-consec | 0.8 | 5 | 14.72 (6.83) | 5 | 12.48 (5.69) | 3 | 20.93 (10.88) | 3 | 20.93 (11.45) |
|  | Non-consec | 0.6 | 3 | 25.41 (10.94) | 3 | 22.27 (9.78) | 2 | 28.39 (14.83) | 2 | 27.52 (14.29) |
|  | Consec | 0.8 | 4 | 20.68 (10.57) | 4 | 18.29 (9.54) | 3 | 23.85 (15.18) | 3 | 23.27 (14.81) |
|  | Consec | 0.6 | 3 | 27.17 (13.74) | 3 | 24.15 (11.83) | 2 | 30.17 (16.83) | 2 | 30.01 (18.65) |
|  |  |  |  |  |  |  |  |  |  |  |
| Weekday  Sleep Variability | Non-consec | 0.8 | 14 | 5.72 (2.71) | 14 | 5.28 (3.41) | 14 | 5.53 (3.05) | 15 | 5.01 (3.23) |
|  | Non-consec | 0.6 | 9 | 9.94 (4.62) | 9 | 9.17 (5.80) | 9 | 9.60 (5.59) | 10 | 9.34 (6.08) |
|  | Consec | 0.8 | 14 | 5.83 (3.09) | 15 | 4.56 (3.07) | 15 | 4.92 (3.04) | 16 | 4.39 (3.12) |
|  | Consec | 0.6 | 10 | 9.31 (5.34) | 10 | 8.67 (6.04) | 9 | 10.08 (6.48) | 11 | 9.23 (6.59) |
|  |  |  |  |  |  |  |  |  |  |  |
| Weekend  Sleep Variability | Non-consec | 0.8 | 7 | 5.73 (3.08) | 7 | 4.93 (2.92) | 6 | 7.85 (5.54) | 6 | 7.75 (5.14) |
|  | Non-consec | 0.6 | 5 | 12.99 (7.38) | 5 | 11.25 (5.79) | 5 | 11.32 (8.30) | 5 | 11.32 (8.37) |
|  | Consec | 0.8 | 6 | 9.28 (4.91) | 6 | 8.29 (4.99) | 6 | 7.68 (6.63) | 6 | 7.92 (5.56) |
|  | Consec | 0.6 | 5 | 13.20 (7.65) | 5 | 11.22 (6.27) | 4 | 15.09 (11.72) | 4 | 15.01 (10.95) |
|  |  |  |  |  |  |  |  |  |  |  |

Means (SDs) are presented for weekly and monthly time windows for the minimum number of days obtained from ICC analyses for 0.8 and 0.6 reliability threshold. Results are partitioned into weekday-only and weekend-only analyses.
